# Novel neuroprotective peptides in the venom of the solitary scoliid wasp *Scolia decorata ventralis*

**DOI:** 10.1590/1678-9199-JVATITD-2020-0171

**Published:** 2021-06-11

**Authors:** Carlos Alberto-Silva, Fernanda Calheta Vieira Portaro, Roberto Tadashi Kodama, Halyne Queiroz Pantaleão, Marisa Rangel, Ken-ichi Nihei, Katsuhiro Konno

**Affiliations:** 1 Natural and Humanities Sciences Center, Experimental Morphophysiology Laboratory, Federal University of ABC (UFABC), São Bernardo do Campo, SP, Brazil.; 2 Immunochemistry Laboratory, Butantan Institute, São Paulo, SP, Brazil.; 3 Faculty of Agriculture, Utsunomiya University, Utsunomiya, Tochigi, Japan.; 4 Institute of Natural Medicine, University of Toyama, Toyama, Toyama, Japan.

**Keywords:** Comprehensive analysis, LC-ESI-MS, Solitary wasp, Venom, Neuroprotective peptide

## Abstract

**Background:**

Solitary wasp venoms may be a rich source of neuroactive substances, since their venoms are used for paralyzing preys. We have been exploring bioactive constituents of solitary wasp venoms and, in this study, the component profile of the venom from a solitary scoliid wasp, *Scolia decorata ventralis*, was investigated through a comprehensive analysis using LC-MS. Two peptides were synthesized, and their neuroprotective properties were evaluated.

**Methods:**

A reverse-phase HPLC connected to ESI-MS was used for LC-MS analyses. Online mass fingerprinting was performed from TIC, and data-dependent tandem mass spectrometry gave the MS/MS spectra. The sequences of two major peptide components were determined by MALDI-TOF/TOF MS analysis, confirmed by solid phase synthesis. Using the synthetic peptides, biological activities were assessed. Cell integrity tests and neuroprotection analyzes using H_2_O_2_ as an oxidative stress inducer were performed for both peptides.

**Results:**

Online mass fingerprinting revealed that the venom contains 123 components, and the MS/MS analysis resulted in 33 full sequences of peptide components. The two main peptides, α-scoliidine (DYVTVKGFSPLR) and β-scoliidine (DYVTVKGFSPLRKA), present homology with the bradykinin C-terminal. Despite this, both peptides did not behave as substrates or inhibitors of ACE, indicating that they do not interact with this metallopeptidase. In further studies, β-scoliidine, but not α -scoliidine, showed protective effects against oxidative stress-induced neurotoxicity in PC12 cells through integrity and metabolism cell assays. Interestingly, β-scoliidine has the extension of the KA dipeptide at the C-terminal in comparison with α-scoliidine.

**Conclusion:**

Comprehensive LC-MS and MS/MS analyses from the *Scolia decorata ventralis* venom displayed the component profile of this venom. β-scoliidine showed an effective cytoprotective effect, probably due to the observed increase in the number of cells. This is the first report of solitary wasp venom peptides showing neuroprotective activity.

## Background

Arthropod venoms are a rich source of bioactive and potentially useful peptides [1]. In particular, spider and scorpion venoms have a variety of neuroactive peptides acting on ion channels and receptors. They have been used for studying neuronal functions and some of them may be useful for medical and agricultural application [2,3]. It is the case for Hymenopteran insect venoms from social bees and wasps such as honeybees and hornets. Their venoms are used for defending their nests, larvae and themselves from predators and are composed of a variety of peptides and proteins [4].

We are interested in solitary wasps and have researched their venom components over the past few decades. Solitary wasps use their venoms to capture and paralyze their prey, insects and spiders, and feed their larvae with paralyzed prey [5]. Accordingly, solitary wasp venoms may have neuroactive substances as major components. In fact, we found sodium-channel blocking neurotoxins, α- and β-pompilidotoxins, in spider wasp venoms [6,7], and ASIC channel inhibitor, Sa12b, in sphecid wasp venoms [8]. Additionally, bradykinin-related peptides [9] and antimicrobial peptides [10] were also found. Thus, our studies of solitary wasp venom components revealed that the venoms contain not only neuroactive peptides but also a variety of bioactive peptides [11].

The first study of solitary wasp venoms was reported in 1987. Two kinins, threonine^6^-bradykinin (Thr^6^-BK) and megascoliakinin, were isolated and characterized from the venoms of the European scoliid wasps *Colpa interrupta* and *Megascolia flavifrons*, respectively [12,13]. These kinins irreversibly block the synaptic transmission of the nicotinic acetylcholine receptor (nAChR) in the prey central nervous system [13,14]. Thr^6^-BK may be a common component of solitary scoliid wasp venoms, since we identified this peptide as a major component in three scoliid wasp venoms found in Japan [15]. Thr^6^-BK is also present in the venom of the spider wasp *Cyphononyx dorsalis* (*Cyphononyx fulvognathus*), which contains three other bradykinin-related peptides: cyphokinin, fulvonin and Cd-146 [9].

In this study, we focused on another Japanese species of solitary scoliid wasp, *Scolia decorata ventralis*, belonging to Scoliidae family and *Scolia* genus, and preying beetle larva in the ground. Comprehensive LC-MS and MS/MS analyses of the crude venom extract depicted the component profile of this venom, and the two major peptide components, α-scoliidine and β-scoliidine, were bradykinin-related peptides. In neuroprotective assays, β-scoliidine showed an effective cytoprotective effect, probably due to the observed increase in the number of cells. This is the first report on solitary wasp venom peptides showing neuroprotective activity.

## Methods

### Materials

All chemicals used in the present study were of analytical reagent grade (purity higher than 95%) and purchased from Calbiochem-Novabiochem Corporation (USA), Gibco BRL (New York, USA), Fluka Chemical Corp. (Buchs, Switzerland) or Sigma-Aldrich Corporation (MO, USA). The ACE I from rabbit lung (EC 3.4.15.1) from Sigma-Aldrich. For the reverse phase chromatography, acetonitrile and TFA were acquired from J.T. Baker. The FRET substrate Abz-FRK(Dnp)P-OH (used by ACE-I assays) were provided by Prof. Adriana Carmona, from the Department of Biophysics of UNIFESP-EPM, São Paulo, Brazil.

*Wasp collection*

Five female wasp individuals of *Scolia decorata ventralis* were collected manually by an insect-catching net in Kyoto, Japan, in August 2010. The venom sacs were dissected under a low temperature anesthetization and extracted with 50% MeCN (acetonitrile) /water. The extracts were lyophilized and stored at -35 ˚C until use. 

*LC-ESI-MS*

The crude venom was analyzed with a LC (Accela 600 Pump, Thermo Scientific) connected with ESI-FTMS (LTQ Orbitrap XL, Thermo Scientific). The lyophilized venom sac extracts were dissolved into 500 ml of water, and from this solution, 10 ml (corresponding to 10% amount of crude venom sac extracts from a single specimen) was subjected to reversed-phase HPLC using CAPCELL PAK C_18_ UG 120, 1.5 x 150 mm (SHISEIDO Co., Ltd., Tokyo, Japan) with linear gradient from 5% to 65% CH_3_CN/H_2_O/0.1% (v/v) formic acid at a flow rate of 200 µL/min over 20 min at 25 °C. ESI-FTMS was operated by Xcalibur^TM^ software (Thermo Scientific) as: capillary voltage, +4.6 kV; capillary temp., 350 °C; sheath and aux gas flow, 50 and 30, respectively (arbitrary units); resolution, 5 ppm. MS/MS spectra were obtained by data dependent MS/MS mode (two most intense peaks by HCD) and the obtained spectra were manually analyzed to give peptide sequences, which were confirmed by MS-Product in ProteinProspector program (http://prospector.ucsf.edu/prospector/cgi-bin/msform.cgi?form=msproduct).

*MALDI-TOF MS*

MALDI-TOF MS spectra were acquired on an Autoflex TOF/TOF mass spectrometer (Bruker Daltonics, Yokohama, Japan) equipped with 337 nm pulsed nitrogen laser under reflector mode. The resolution and accuracy of MS were 18000 full width at half maximum (*m/z* 3000) and 10 ppm, respectively. The accelerating voltage was 20 kV. Matrix, α-cyano-4-hydroxycinnamic acid (Aldrich), was prepared at a concentration of 10 mg/mL in 1:1 CH_3_CN/ 0.1% (v/v) TFA. External calibration was performed with [Ile7]-angiotensin III (*m/z* 897.51, monoisotopic, Sigma) and human ACTH fragment 18-39 (*m/z* 2465.19, monoisotopic, Sigma). The sample solution (0.5 µL) dropped onto the MALDI sample plate was added to the matrix solution (0.5 µL) and allowed to dry at room temperature. For TOF/TOF measurement, argon was used as a collision gas and ions were accelerated at 19 kV. The series of *b* and *y* ions were afforded, which enabled identification of whole amino acid sequence by manual analysis.

*Peptide synthesis*

The peptides were synthesized using Fmoc chemistry by GenScript (Nanjing, Jiangsu, China). The crude products were purified by RP-HPLC with a preparative C18 column, and the purity and molecular weight of the final peptides were verified by HPLC and MS.

*Kinetic analyses with α-scoliidine and β-scoliidine as NEP and ACE inhibition*

Experiments were performed using different concentrations of both peptides with ACE and the Abz-FRK(Dnp)P-OH as substrate. The assays used 7.5 ng of the peptidase and the substrate was added in a 100 mM Tris-HCl buffer containing 50 mM NaCl, 10 μM ZnCl_2_, pH 7.0 (final volume of 100 µL). Three FRET substrate concentrations were used (2 μM, 4 μM and 8 μM), and were incubated with three concentrations of both peptides (20 μM, 30 μM and 50 μM). Controls without the peptides were also performed in all assays. The reaction was monitored during 15 minutes on fluorimeter (Victor 3 - Perkin-Elmer) and the results were analyzed on GraFit 3.0 from Erithacus Software. All assays were performed in triplicate.

*α-scoliidine and β-scoliidine stability tests*

Both peptides (30 µM) were incubated separately with ACE (7.5 n) at 37°C for four hours. Samples containing only the synthetic peptides were used as negative control. After incubation, samples were analyzed by reverse phase chromatography on HPLC (Prominence, Shimadzu), using a Shim-pack VP-ODS C-18 column (4.6× 150 mm). Solvents used were 0.1% (v/v) TFA in water (solvent A), and acetonitrile plus solvent A (9:1) as solvent B. Separations were performed at a flow rate of 1 mL/min and a 10-60% gradient of solvent B over 20 min. In all cases, elution was followed by the measurement of ultraviolet absorption (214 nm).

*PC12 cell culture*

Neuronal cell line (PC12) was used to evaluate the neuroprotective effects of α-scoliidine and β-scoliidine against oxidative stress *in vitro*. PC12 cell is derived from a transplantable rat pheochromocytoma and was purchased from American Type Culture Collection (USA) (ATCC® CRL-1721™). Cells were routinely cultured in DMEM medium (Sigma-Aldrich) supplemented with 10% fetal bovine serum (Gibco, Waltham, USA), 1% (v/v) of 10000 U/mL penicillin, 10 mg/mL streptomycin and 25 μg/mL amphotericin B solution (Sigma-Aldrich) at 37ºC in an atmosphere of 5% CO_2_ and 95% air (Water Jacketed CO_2_ Incubator, Thermo Scientific). At 80% confluence, cells were passaged using trypsin-EDTA solution [0.05% (m/v) trypsin and 0.02% (m/v) EDTA]. Culture medium was replaced every 2-3 days.

*Cell toxicity studies*

PC12 cells were seeded into 96-well plates (Nest Biotechnology, Rahway, USA), at 2.0 × 10^4^ cells per well, and were treated with different concentrations (0.1 to 10 µM) of α-scoliidine and β-scoliidine in a final volume of 0.15 mL. The plate was incubated at 37°C for 3, 6, 12, 24, and 48 h. For each concentration and time course studied, there were control and DMSO groups, which represent untreated cells (only one equal volumes of the culture medium) and treated with DMSO 2.5% (v/v) diluted in the medium culture, respectively. The cytotoxic effects of both compounds were determined using crystal violet assay, according to Feoktistova et al. [16]. Briefly, the medium was aspirated, and the cells were washed twice in a gentle stream of tap water. Then, 50 μL of a 0.5 % (v/v) crystal violet staining solution was added to each well, and incubated for 20 min at room temperature on a bench rocker with a frequency of 20 oscillations per min. The plate was washed four times and air-dried for at least 2 h at room temperature. Subsequently, methanol (200 μL) was added to each well, and the plate was incubated with its lid on for 20 min at room temperature on a bench rocker with a frequency of 20 oscillations per min. After, the absorbance of the sample was measured at 570 nm using a SpectraMax reader (Molecular Devices, CA, USA). The cytotoxicity was expressed as percentage (%) of cell integrity relative to control cells (100%).

*Neuroprotective assay*

PC12 cells were seeded at 2 x 10^4^ cells/well in a 96-well plate (Nest Biotechnology, Rahway, USA) for 24 h, and then two neuroprotective assays were evaluated: 1) Cells were pre-treated with α-scoliidine or β-scoliidine (1 µM) at 37°C for 22 h and, after the mediums were replaced containing the peptide and H_2_O_2_ (0.6 mM) and incubated for 2 h more; 2) Cells were pre-treated with α-scoliidine or β-scoliidine (1 µM) for 4 h at 37°C and, after the mediums were replaced containing peptide and H_2_O_2_ (0.6 mM) and incubated for 20 h more. The control, α-scoliidine and β-scoliidine groups (without H_2_O_2_) were incubated under the same conditions. Next, all experimental groups were analyzed by the crystal violet assay.

*Mitochondrial metabolism evaluation*

The effects of α-scoliidine or β-scoliidine on the mitochondrial metabolism of PC12 cells were estimated using 3-(4,5-dimethylthiazol-2-yl)-2,5-diphenyltetrazolium bromide (MTT) reduction assay, as previously described [17,18]. Briefly, cells were placed on 96-well plates (Nest Biotechnology, Rahway, USA) at 2.0×10^4^/well, and treated as described above for neuroprotection assays - protocol 2. In addition, to the experimental groups mentioned above, two more groups were included in which cells were treated with L-N^Ω^-Nitroarginine methyl ester (L-Name; 1 mM) and Bradykinin (Bk; 0.3 µM) in the presence or absence of H_2_O_2_. After treatments, cells were treated with 0.5 mg/mL MTT in the same medium culture for 3 h at 37°C. The resulting formazan produced by both cell treatments were dissolved by addition of DMSO. The amount of MTT formazan dissolved in DMSO was determined by measuring absorbance with a microplate reader (Spectramax M3 multi-mode, Molecular Devices, CA, EUA) at 540 nm. Data were expressed as the mean ± SD of mitochondrial metabolism percentage in relation to the control.

*Statistical analyses*

All data are presented as mean ± SD of three independent experiments (n = 3) in triplicate. Data were analyzed using one-way analysis of variance (ANOVA) for between-groups comparisons followed by a Tukey’s post-hoc test for multiple comparisons. Values of *p* < 0.05 were considered to be statistically significant. The analyses were performed using GraphPad Prism 6.0 software (GraphPad Software, Inc., La Jolla, CA).

## Results

### Online mass fingerprinting by LC-MS

**Figure 1:**
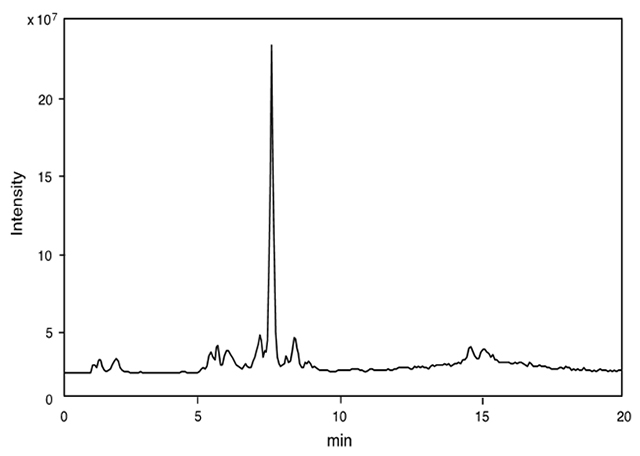
TIC profile from LC-ESI-MS of crude venom extracts of *Scolia decorata ventralis*. An amount of 10% of crude venom extracts of a single specimen was applied to reverse-phase HPLC using CAPCELL PAK C_18_ (1.5 x 150 mm) with linear gradient of 5-65% CH_3_CN/H_2_O/0.1% (v/v) formic acid over 20 min at flow rate of 200 µL/min.

The crude venom extract was first subjected to LC-ESI-MS in order to verify the component profile: number of components and its molecular mass determination. The TIC profile is shown in Figure 1. The volume of the sample solution never exceeded the amount of 10% of crude venom, being a single specimen sufficient for LC-ESI-MS analysis (mass fingerprinting and peptide sequencing). Online mass fingerprint was prepared from TIC by “virtual fractionation”, collecting MS spectra from certain range of retention time (fractions), and then, the molecular mass was analyzed in each fraction. The results are summarized in Table 1. The TIC profile seems to be simple, but comprehensive analysis resulted to find a large number of components in this venom extract. A total of 123 molecular mass in *m/z* range of 90-9500 were obtained from 17 virtual fractions. The low molecular mass components (33 components, *m/z* 90-300) may be free amino acids, biogenic amines and nucleic acids, and those of *m/z* range 300 - 9500 should be peptides (91 components), in particular, those of *m/z* 500-2000 (68 components) accounts for 75% of the peptide components, implying that major components in this venom are relatively small peptides.

**Table 1:** Online mass fingerprinting of crude venom extract from *Scolia decorata ventralis* by LC-ESI-MS.

Fr	RT	(M+H)^+^
1	0.8-1.5	90.055, 106.050, 112.081, 116.070, 118.086, 120.066, 133.061, 134.045, 146.165, 147.076, 147.113, 148.060, 156.077, 175.119, 203.223, 258.110, 324.159
2	1.5-2.0	132.102, 138.091, 150.058, 154.086, 182.081, 183.065, 244.093, 245.077, 268.104, 348.070, 428.037, 664.116, 2684.460
3	2.0-3.0	166.086, 177.102, 269.088, 552.179, 608.374, 799.469, 817.452, 934.176, 1041.627
4	3.0-4.0	609.360, 682.377, 814.453, 1062.641, 7359.757
5	4.0-4.8	205.097, 666.382, 1076.575, 1200.648, 1249.558, 1362.701
6	4.8-5.4	723.403, 818.487, 920.473, 1102.673, 1344.764, 1365.749, 4988.838, 5188.982
7	5.4-6.4	619.356, 793.384, 875.509, 903.541, 1274.674, 1302.790, 1477.755, 1639.808, 6512.873, 9307.706, 9509.785
8	6.4-7.3	676.378, 1110.543, 1216.669, 1267.797, 1465.854, 1783.960, 1946.012, 2077.142, 3464.720, 4117.402, 4231.401, 6220.708
9	7.3-7.7	813.450, 1266.721, 1302.790, 1580.880
10	7.7-8.2	1594.896, 1638.886, 1660.837, 1719.990, 3564.050
11	8.2-8.6	928.477, 1112.562, 1381.748, 1754.896, 7191.971, 7379.036
12	8.6-9.2	1005.608, 1083.651, 1319.706, 1535.857, 1652.864, 1718.873, 1732.945, 1801.042, 4553.337
13	9.2-10.0	930.562, 1117.636, 1120.672, 1225.647, 2147.097
14	10.0-11.0	695.424, 1014.630, 1113.699, 1234.716, 1363.758
15	11.0-12.0	1137.657, 1552.931, 2315.384, 2516.458, 4436.449, 4638.414
16	12.0-13.0	1290.735, 4071.333
17	13.0-14.0	2113.289

### Identification of small molecules (amino acids, biogenic amines and nucleic acids)

A total of 33 small molecules (17 amino acids, 7 biogenic amines, 9 nucleic acids) were identified as summarize in Tables 2-4. The identification was made by the previously reported method [19]: elemental composition analysis of molecular ion (M+H)^+^ with error limit of 0.005 Da, and in some cases, concomitant detection of iminium ion and deamination (-NH_3_) peak. For nucleic acids (AMP, ADP and NAD), MS/MS spectra were obtained by data dependent MS/MS measurement, which confirmed the structure of these compounds. Usually, analysis of these small molecules by reverse-phase HPLC is not easy because they are eluted as a complex mixture in the solvent front portion. Accordingly, derivatization or special HPLC conditions are needed to analyze [20-22]. In contrast, the method utilizing LC-MS, as previously reported and shown here, is easy and advantageous that it can be applicable for small molecule analysis of any animal venom.

**Table 2:** Amino acids in the crude venom extract from *Scolia decorata ventralis* by LC-ESI-MS.

RT (min)	Intensity x 10^4^	[M + H]^+^ *m/z*	Elemental composition	Iminium ion *m*/*z*	Elemental composition	Compound
1.11	6	147.113	C_6_H_15_N_2_O_2_	-		Lysine
1.25	11	90.055	C_3_H_7_NO_2_	-		Alanine
	2	106.050	C_3_H_8_NO_3_	-		Serine
	8	120.065	C_4_H_10_NO_3_	-		Threonine
	7	133.061	C_4_H_9_N_2_O_3_	-		Asparagine
	24	134.045	C_4_H_8_NO_4_	-		Aspartic acid
	200	147.076	C_5_H_11_N_2_O_3_	-		Glutamine
	60	156.077	C_6_H_10_N_3_O_2_	-		Histidine
	180	175.119	C_6_H_15_N_4_O_2_	-		Arginine
1.32	900	116.070	C_5_H_10_NO_2_	70.065	C_4_H_8_N	Proline
	80	118.086	C_5_H_12_NO_2_	-		Valine
	150	148.060	C_5_H_10_NO_4_	-		Glutamic acid
1.67	35	150.058	C_5_H_12_NO_2_S	-		Methionine
1.81	300	132.102	C_6_H_14_NO_2_	86.096	C_5_H_12_N	L/I
1.88	80	182.081	C_9_H_12_NO_3_	-		Tyrosine
2.76	180	166.086	C_9_H_12_NO_2_	-		Phenylalanine
4.79	26	205.097	C_11_H_13_N_2_O_2_	-		Tryptophan

**Table 3:** Biogenic acines in the crude venom extract from *Scolia decorata ventralis* by LC-ESI-MS.

RT (min)	Intensity x 10^4^	[M + H]^+^ *m/z*	Elemental composition	Deamination *m*/*z*	Elemental composition	Compound
1.04	550	112.087	C_5_H_10_N_3_	95.060	C_5_H_7_N_2_	Histamine
	10	146.165	C_7_H_20_N_3_	-		Spermidine
	25	203.223	C_10_H_27_N_4_	-		Spermine
1.67	11	154.086	C_8_H_12_NO_2_	137.060	C_8_H_9_O_2_	Dopamine
1.88	1600	138.091	C_8_H_12_NO	121.065	C_8_H_9_O	Tyramine
2.32	6	177.102	C_10_H_13_N_2_O	160.076	C_10_H_10_NO	Serotonin
3.49	4	146.117	C_7_H_16_NO_2_	-		Acetylcholine

**Table 4:** Nucleic acids in the crude venom extract from *Scolia decorata ventralis* by LC-ESI-MS.

RT (min)	Intensity x 10^4^	[M + H]^+^ *m/z*	Elemental composition	Compound
1.32	200	258.110	C_10_H_16_N_3_O_5_	Thymidine
1.53	60	244.093	C_9_H_14_N_3_O_5_	Cytidine
	16	348.070	C_10_H_15_N_5_O_7_P	Adenosine monophosphate
	13	428.037	C_10_H_16_N_5_O_10_P_2_	Adenosine diphosphate
1.60	50	664.116	C_21_H_28_N_7_O_14_P_2_	NAD
1.74	12	245.077	C_9_H_13_N_2_O_6_	Uridine
1.81	2000	268.104	C_10_H_14_N_5_O_4_	Adenosine
1.98	30	284.099	C_10_H_14_N_5_O_5_	Guanosine
2.10	150	269.088	C_10_H_13_N_4_O_5_	Inosine

### Peptide sequencing by LC-MS/MS analysis

Data dependent MS/MS measurement with LC-MS afforded MS/MS spectra from 60 peptide molecules. Manual sequence analysis of these MS/MS spectra revealed the full sequence of 33 peptides, and the rest of the 27 peptides were only partially sequenced (data not shown). The analyzed full sequences are shown in Table 5.

The two most intense peaks in Fr. 9 and 11 contained the major peptides β-scoliidine (*m/z* 1580.880, DYVTVKGFSPLRKA) and α-scoliidine (*m/z* 1381.748, DYVTVKGFSPLR), respectively. They are different each other only at the C-terminal: β-scoliidine has the dipeptide KA at the C-terminal of β-scoliidine. L (leucine) at the position 11 of both peptides was determined by MALDI TOF/TOF analysis. The MALDI TOF/TOF spectra of both peptides showed *Wa* ion peak: β-scoliidine, *m/z* 482.2 (*W*_*a4*_ ); α-scoliidine *m/z* 229.0 (*W*_*a2*_), which clearly showed that the residue at these positions are both L (leucine), not I (isoleucine). The solid-phase synthesis of these peptides, and the HPLC and MS/MS comparisons of the synthetic specimens with the natural peptides finally corroborated the sequences. These peptides can belong to bradykinin-related peptides because the sequences are homologous to those of known bradykinin-related peptides, in particular, to Cd-146 (Table 6).

The analyzed full sequences can be classified according to homology and similarity as shown in Table 7. The major class is bradykinin-related peptides including α-scoliidine and β-scoliidine, and the rest of the classes may be novel peptides because all these have no homology or similarity to any known peptides. Within each class, the longest sequence can be “parent peptide”, and others are truncated peptides from both N- and C-terminus of the parent peptide. Seemingly, these truncated peptides are cleavage products of the parent peptide in some way, but it is not sure whether they are originally contained in the venom or not.

**Table 5:** Peptide sequences analyzed from MS/MS spectra.

Fr	RT	Intensity x 10^3^	MSMS *m/z*	charge	(M+H)^+^	Sequence
3	2.20	105	400.238	2+	799.469	RLFAHR
	2.86	19	409.230	2+	817.452	TSVERQV-NH_2_
	2.88	42	304.692	2+	608.374	YVTVK-NH_2_
4	3.02	13	354.886	3+	1062.641	AFLEARTKK-NH_2_
	3.66	170	305.184	2+	609.360	YVTVK
	4.11	50	333.695	2+	666.382	YVTVKG
	4.25	750	359.530	3+	1076.575	RLFAHRNY
6	4.84	9	307.496	3+	920.473	LFAHRNY
	4.96	180	455.921	3+	1365.749	SESAFLEARTKK-NH_2_
	5.04	240	362.206	2+	723.403	DYVTVK-NH_2_
	5.16	63	448.926	3+	1344.764	GKSGNPFSKPVVK
	5.26	6	368.230	3+	1102.673	VKGFSPLRKA
7	5.60	42	397.196	2+	793.384	GKSGNPFS
	5.73	30	425.563	3+	1274.674	TPRLFAHRNY
	6.00	77	310.182	2+	619.356	FSPLR
	6.09	10	301.852	3+	903.541	VKGFSPLR
	6.21	21	434.935	3+	1302.790	VTVKGFSPLRKA
8	6.54	240	406.228	3+	1216.668	GKSGNPFSKPVV
	6.81	500	489.290	3+	1465.854	YVTVKGFSPLRKA
	7.11	15	555.775	2+	1110.543	SESAFLEART
9	7.43	28000	527.632	3+	1580.880	DYVTVKGFSPLRKA (β-scoliidine)
	7.52	4500	434.935	3+	1302.790	VTVKGFSPLRKA
	7.62	130	407.229	2+	813.450	YVTVKGF
	7.68	330	422.912	3+	1266.721	YVTVKGFSPLR
11	8.28	570	464.742	2+	928.477	DYVTVKGF
	8.38	1700	461.254	3+	1381.748	DYVTVKGFSPLR (α-scoliidine)
	8.43	150	556.785	2+	1112.562	DYVTVKGFSP
12	9.01	360	542.329	2+	1083.651	DVPRLLTSLA-NH_2_
13	9.35	7	559.322	2+	1117.636	DVLAFRVDLA-NH_2_
	9.66	6	374.229	3+	1120.672	SFTDLLKGLK-NH_2_
14	10.16	5	455.258	3+	1363.758	ENSFTDLLKGLK-NH_2_
	10.29	8	412.244	3+	1234.716	NSFTDLLKGLK-NH_2_
	10.43	95	348.216	2+	695.424	LRFLF
	10.94	24	557.354	2+	1113.699	LTLTRDVLLA-NH_2_

**Table 6:** Bradykinin-related peptides in solitary wasp venoms.

Peptides	Sequence
Thr^6^-Bradykinin	RPPGFTPFR
Megascoliakinin	RPPGFTPFRKA
Cyphokinin	DTRPPGFTPFR
Fulvonin	SIVLRGKAPFR
Cd-146	SETGNTVTKGFSPLR
α-Scoliidine	DYVTVKGFSPLR
β-Scoliidine	DYVTVKGFSPLRKA

**Table 7:** Classification of the peptide sequences.

RT	Intensity x 10^3^	(M+H)^+^	Sequence
6.00	77	619.356	FSPLR
6.09	10	903.541	VKGFSPLR
5.26	6	1102.673	VKGFSPLRKA
7.52	4500	1302.790	VTVKGFSPLRKA
3.66	170	609.360	YVTVK
2.88	42	608.374	YVTVK-NH_2_
4.11	50	666.382	YVTVKG
7.62	130	813.450	YVTVKGF
7.68	330	1266.721	YVTVKGFSPLR
6.81	500	1465.854	YVTVKGFSPLRKA
5.04	240	723.403	DYVTVK-NH_2_
8.28	570	928.477	DYVTVKGF
8.43	150	1112.562	DYVTVKGFSP
8.38	1700	1381.748	DYVTVKGFSPLR (α-scoliidine)
7.43	28000	1580.880	DYVTVKGFSPLRKA (β-scoliidine)
2.20	105	799.469	RLFAHR
4.84	9	920.473	LFAHRNY
4.25	750	1076.575	RLFAHRNY
5.73	30	1274.674	TPRLFAHRNY
7.11	15	1110.543	SESAFLEART
3.02	13	1062.641	AFLEARTKK-NH_2_
4.96	180	1365.749	SESAFLEARTKK-NH_2_
5.60	42	793.384	GKSGNPFS
6.54	240	1216.668	GKSGNPFSKPVV
5.16	63	1344.764	GKSGNPFSKPVVK
9.66	6	1120.672	SFTDLLKGLK-NH_2_
10.29	8	1234.716	NSFTDLLKGLK-NH_2_
10.16	5	1363.758	ENSFTDLLKGLK-NH_2_
10.43	95	695.424	LRFLF
2.86	19	817.452	TSVERQV-NH_2_
10.94	24	1113.699	LTLTRDVLLA-NH_2_
9.01	360	1083.651	DVPRLLTSLA-NH_2_
9.35	7	1117.636	DVLAFRVDLA-NH_2_

### Enzymatic assays

Both α-scoliidine and β-scoliidine were unable to inhibit the catalytic activity of ACE. In addition, both peptides were not cleaved by the metallopeptidase, indicating that they do not interact with the studied enzyme (Table 8).

**Table 8:** Hydrolyses of α-scoliidine and β-scoliidine by human neprilysin (NEP) and angiotensin-converting enzyme (ACE).

	Inhibition (%)		Cleavage (%)	
	ACE	NEP	ACE	NEP
α-scoliidine	< 0.01	< 0.01	< 0.01	< 0.01
β-scoliidine	< 0.01	< 0.01	< 0.01	< 0.01

### Neurotoxicity of α-scoliidine and β-scoliidine

**Figure 2:**
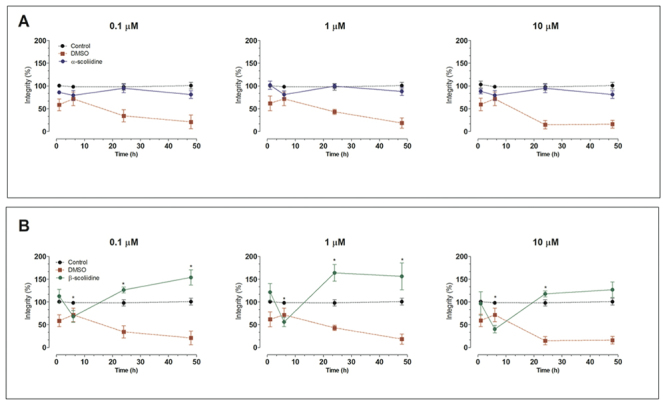
Effect of α-scoliidine and β-scoliidine on cell viability in PC12 cells. Cells treated with either **(A)** α-scoliidine or **(B)** β-scoliidine in concentrations varying from 0.1 to 10 μM for 3, 6, 12, 24, and 48 h. Control and DMSO groups represent cell without treatment and treated with DMSO 2.5%, respectively. Values are expressed as mean ± standard deviation from three independent experiments in triplicate and analyzed by one-way ANOVA followed by Tukey’s post-test. *p< 0.05 in relation to the control group.

The cytotoxic effects of both peptides were assessed using the PC12 cell line as illustrated in Figure 2. The α-scoliidine did not show any significant cytotoxicity (p > 0.05) in all dose concentrations, and at the times tested when compared to the untreated cell (control) or DMSO (Figure 2A). Interestingly, β-scoliidine decreased cell integrity after 6 h of treatment in all concentration tested (Figure 2B). However, after 24 and 48 h of treatment with this peptide, cell integrity increased significantly (p < 0.05), especially at 0.1 and 1 µM, compared to the control group. DMSO decreased the cell integrity after 24 and 48 h compared to the control group, which is in accordance with the literature [23]. The α-scoliidine and β-scoliidine at 1 µM for 24 h of treatments were chosen to carry out neuroprotective assays.

### Neuroprotective effects against H_2_O_2_-induced oxidative stress

**Figure 3:**
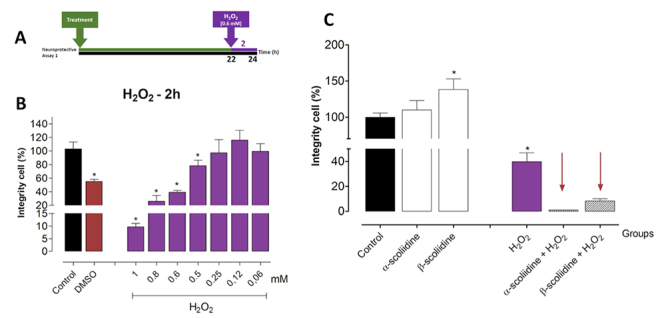
Effect of acute H_2_O_2_-induced oxidative stress on cell viability and neuroprotective evaluation of either α-scoliidine or β-scoliidine in PC12 cells. PC12 cells were seeded at 2 x 10^4^ cells/well in a 96-well plate for 24 h, and then submitted to different treatment. **(A)** Neuroprotective assay scheme where cells were treated with α-scoliidine or β-scoliidine (1 µM) for 22 h at 37°C and, after the medium were replaced containing peptide and H_2_O_2_ (0.6 mM) and incubated for more 2h. **(B)** Dose-response of H_2_O_2_for concentrations varying between 1-0.06 mM for 2 h. **(C)** Protective effects of α-scoliidine or β-scoliidine against oxidative stress-induced neurotoxicity. Values are expressed as mean ± standard deviation from three independent experiments in triplicate and analyzed by one-way ANOVA followed by Tukey’s post-test. *p <0.05 to differences among the control group.

**Figure 4:**
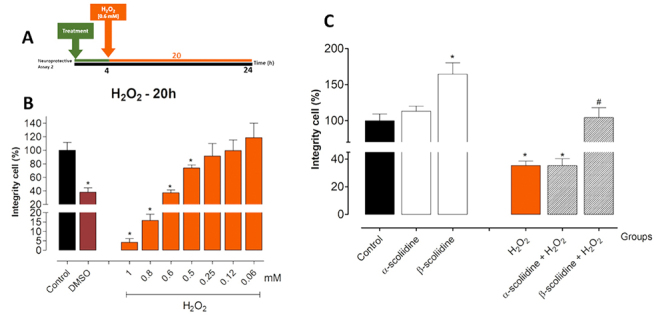
Effect of chronic H_2_O_2_-induced oxidative stress on cell viability and neuroprotective evaluation of either α-scoliidine or β-scoliidine in PC12 cells. PC12 cells were seeded at 2 x 10^4^ cells/well in a 96-well plate for 24 h, and then submitted to different treatment. **(A)** Neuroprotective assay scheme where cells were treated with α-scoliidine or β-scoliidine (1 µM) for 4 h at 37°C and, after the medium were replaced containing peptide and H_2_O_2_ (0.6 mM) and incubated for more 20 h. **(B)** Dose-response of H_2_O_2_for concentrations varying between 1-0.06 mM for 20 h. **(C)** Protective effects of α-scoliidine or β-scoliidine against oxidative stress-induced neurotoxicity. The cell integrity was analyzed by crystal violet protocol. Values are expressed as mean ± standard deviation from three independent experiments in triplicate and analyzed by one-way ANOVA followed by Tukey’s post-test. *p <0.05 to differences among the control group, #*p*< 0.05 when compared with the H_2_O_2_group.

The neuroprotective effects against H_2_O_2_-induced oxidative stress in the presence of α-scoliidine and β-scoliidine were studied in PC12 cells using two experimental conditions of treatments (Figures 3 and 4). In the first assay (Figure 3A), H_2_O_2_ was cytotoxic at concentrations greater than 0.5 mM after 24 h of treatment, showing a dose-response effect. Thus, 0.6 mM of H_2_O_2_ was chosen for subsequent studies (Figure 3B). The α-scoliidine and β-scoliidine (1 μM) pre-treatment did not prevent the reduction of cell integrity, but significantly increased the H_2_O_2_-induced toxicity (Figure 3C). In the second assay (Figure 4A), H_2_O_2_ also showed a dose-response effect after 24 h of treatment, as in the first neuroprotective assay, and 0.6 mM dose was chosen for further studies (Figure 4B). Pre-treatment with β-scoliidine increased the integrity of PC12 cells exposed to oxidative stress compared with to control (H_2_O_2_) (Figure 4C). Besides, cells pre-treated with α-scoliidine did not present significant differences against H_2_O_2_-induced oxidative stress.

### Neuroprotective property on mitochondrial metabolism

**Figure 5:**
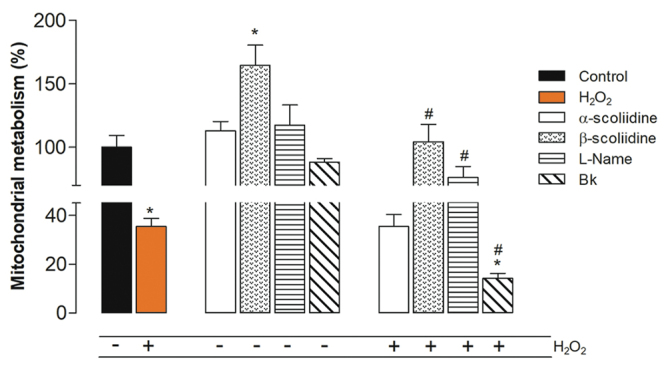
Neuroprotective property of α-scoliidine or β-scoliidine on mitochondrial metabolism of the PC12 cell line against H_2_O_2_-induced oxidative stress. PC12 cells were seeded at 2 x 10^4^ cells/well in a 96-well plate for 24 h, and treated with α-scoliidine or β-scoliidine (1 µM) for 4 h at 37°C and, after the medium were replaced containing peptide and H_2_O_2_ (0.6 mM) and incubated for more 20 h. Mitochondrial metabolism activity was assessed using 3-(4,5-dimethylthiazol-2-yl)-2,5-diphenyltetrazolium bromide (MTT) protocol. Values are expressed as mean ± standard deviation from three independent experiments in triplicate and analyzed by one-way ANOVA followed by Tukey’s post-test. *p <0.05 to differences among the control group. #*p*< 0.05 when compared with the H_2_O_2_group. L-Name: L-N^Ω^-Nitroarginine methyl ester; Bk: Bradykinin.

Neuroprotective properties of α-scoliidine or β-scoliidine on mitochondrial metabolism of the PC12 cell line against H_2_O_2_-induced oxidative stress were investigated in the present study (Figure 5). Cells treated with H_2_O_2_ for 20 h decreased metabolic viability to 32.23 ± 0.96%. Cells pre-treated with α-scoliidine did not prevent the reduction of metabolic viability caused by H_2_O_2._ On the other hand, β-scoliidine attenuated in 50% the H_2_O_2_-induced metabolic viability reduction. The protective properties against H_2_O_2_-induced cytotoxicity of L-Name (1 mM) and the Bk (0.30 μM) were also evaluated in our study. L-Name pre-treatment prevented the reduction of metabolic viability. In contrast, Bk decreased the metabolic viability compared with the H_2_O_2_ group.

## Discussion

In this study, we have first analyzed the component profile of the crude venom extract of *Scolia decorata ventralis*, a solitary scoliid wasp inhabiting in Japan, by using LC-ESI-MS and MS/MS. It revealed that this venom contained 123 components and a majority of them are small peptides. The peptide sequences were further analyzed by manual analysis of their MS/MS spectra, which led to the determination of full sequence of 33 peptides. Among them, two major peptides, α- and β-scoliidine, were thought to be bradykinin-related peptides due to the sequence similarity to bradykinin-related peptides from solitary wasp venoms. Many of the minor peptides are related to and truncated form of these scoliidine peptides. It is not sure whether they are constitutive of the venom or degradation products of the scoliidine peptides. In any case, they are of interest in viewpoint of structure-activity relationship, which may be a future study. Other than these bradykinin-related peptides, there found 4 different classes of unique peptide components in this venom. But, their function and role in this venom are not clear since they have no homology or similarity to any known peptides. In addition to the peptides, we identified 33 small molecules (amino acids, biogenic amines and nucleic acids). It was done easily and simply by LC-MS and MS/MS analysis as reported previously [19]. Previous studies reported the presence and function of some of these components in wasp venoms. Two most abundant biogenic amines, histamine and tyramine, were reported to be found in social and solitary wasp venoms, playing a role in pain-producing component [20]. Adenosine is contained in solitary spider wasp venoms [24]. Dopamine is resent in the venom of emerald jewel wasp *Ampulex compressa* and implicated in a unique behavior of its prey, American cockroach [21]. Most of the small molecules contained in this wasp venom would give physiological effect when injected into beetle larvae prey, which is remained to be studied.

Most notably, these results were obtained by using only 10% amount of a single venom content. Among the Hymenopteran insect venoms, solitary wasp venom has not been well documented. One of the reasons why may come from the difficulty of collecting sufficient amount of venom for chemical analysis because of their solitary lifestyle. However, as shown in this and previous study [19], the remarkable progress of mass spectrometry in sensitivity made it possible to perform this type of peptidomic analysis with very minute amount of venom.

Solitary wasp venoms are a rich source of neuroactive substances, as their venoms induce paralysis in their prey, keeping them alive to feed their larvae [25]. The first components characterized in solitary wasp venom were bradykinin-related peptides [11] which block the synaptic transmission of the nAChR [12,14]. Other neurotoxins potentiate synaptic transmission of lobster leg muscle by presynaptic mechanisms, as pompilidotoxins [6,7]. Here, we evaluated, for the first time, the neurotoxicity of α-scoliidine and β-scoliidine, two main peptides characterized in the *Scolia decorata ventralis*’ venom, on the neuronal PC12 cell line. Interestingly, α-scoliidine was not cytotoxic in all concentrations and times tested, in contrast to β-scoliidine, which was cytotoxic after 3 h of treatment in all concentrations tested. Despite that, β-scoliidine increased the number of cells. The most pronounced effect was obtained with the dose of 1 µM, after 24 to 48 h of treatment, suggesting that this peptide increases the cell rate proliferation.

Oxidative stress is a condition in which the balance between the production of reactive oxygen species (ROS) and the level of antioxidants is considerably altered, and has been associated with the progression of different neurodegenerative diseases [26]. These diseases use experimental models, such as animals and cell cultures, to understand how oxidative stress can produce neurotoxic effects [26,27].

The cellular stress model used in this work was based on the H_2_O_2_-induced oxidative stress, which stimulates the excessive production of ROS [27] in neuronal cell lines after acute and chronic treatments. In our study, using PC12 cells, H_2_O_2_ promoted cell death in a dose-dependent manner in both protocols performed, and we adopted the 0.6 mM H_2_O_2_ concentration to reduce ~60% cell viability, as reported in other studies [17,18,28-30]. Then, we investigated the neuroprotective effects of α-scoliidine and β-scoliidine against H_2_O_2_-induced oxidative stress in PC12 cells treated with H_2_O_2_ at 0.6 mM for 2 h or 18 h - acute and chronic treatments, respectively.

The acute treatment was not a satisfactory experimental design, since both scoliidines potentiated cell damage mediated by H_2_O_2_, especially the treatment with β-scoliidine. In the acute treatment, β-scoliidine was neurotoxic and α-scoliidine showed a tendency for neurotoxicity at 6 h of treatment, which could explain the increase of H_2_O_2_-mediated neuronal damage. The phenomenon of synergistic toxicity of H_2_O_2_ effects when two agents are used together, and efficiently potentiate cell damage, has been reported in the literature [31]. Of the several examples of H_2_O_2_ toxicity potentiation by other simple chemicals, the best-known case is observed in immune cells with a mixture of H_2_O_2_ and nitric oxide (NO), during the infectious process [32]. Besides, a few other inorganic molecules also potentiate H_2_O_2_ toxicity in mammalian cells [31]. The enhancement of the intracellular oxidative potential of H_2_O_2_ by ascorbic acid, for example, is also reported [33]. Many cancer cell lines are sensitive to extracellular concentrations of ascorbic acid, which are harmless to normal cells. This sensitivity is alleviated by extracellular catalase, indicating that ascorbic acid, associated with H_2_O_2_ toxicity, is the underlying cause of its sensitivity [34]. For these reasons, further studies are needed to explain the potentiating effects of H_2_O_2_ toxicity in the presence of scoliidines.

Neurogenesis and neuroprotection might be stimulated by Bk, being of great interest in clinical applications following brain injury [35]. Bk simultaneously reduces the rate of proliferation and the neuronal enrichment of rat embryonic telencephalon neural precursor cells [36]. In the present study, Bk changed the proliferation rate of PC12 cells, reducing the metabolic viability at 24 h of treatment compared with the control and H_2_O_2_ groups. The two main peptides identified from the *Scolia decorata ventralis* venom were α-scoliidine (DYVTVKGFSPLR) and β-scoliidine (DYVTVKGFSPLRKA) which present homology with the bradykinin C-terminal (RPPGFSPFR), suggesting, therefore, similar activities. For these reasons, both synthetic peptides have been evaluated as possible inhibitors or substrates for ACE, but negative results have shown that scoliidines do not interact with this metallopeptidase. Neuroprotection studies indicated that α-scoliidine, which is similar to the Bk (GFSPFR), did not alter cell proliferation, but both peptides showed different protective effects against neurotoxicity induced by oxidative stress in the PC12 cell.

A neuroprotective compound can diminish the molecular and cellular ROS-mediated damages, preventing or attenuating the progression of the disease and its secondary consequences [26,35]. Studies reported that small structural differences in bioactive peptides promoted remarkable functional differences [6,11,17,18]. Bradykinin-potentiating peptides (BPPs) from the *Bothrops jararaca* snake venom were described as neuroprotective against H_2_O_2_-induced oxidative stress in human neuroblast-like cell line SH-SY5Y [17,18]. BPP-11e (<EARPPHPPIPP) and BPP-AP (<EARPPHPPIPPAP), for example, are not good neuroprotective peptides against the H_2_O_2_-induced oxidative stress [18] in contrast to the BPP-10c (<ENWPHQIPP) [17]. This fact was also observed in the present study, where small structural differences between the two scoliidines resulted in significant differences in biological activities. That is, β-scoliidine demonstrated neuroprotective effect against the H_2_O_2_-induced damage, preserving neuronal cell integrity and mitochondrial metabolism in chronic treatment, but not α-scoliidine. It is very interesting to note that the β-scoliidine effects were dependent on the presence of only two more amino acid residues (KA) in the C-terminal on its primary molecular sequence. Besides, β-scoliidine also increased cell proliferation in contrast to α-scoliidine. Previous works have shown that amyloid-β peptide (Aβ) isoforms found in amyloid plaques of brains with Alzheimer’s disease can affect differentiation and proliferation of rat or mouse neural progenitor cells [37,38]. Specifically, Aβ 42 peptide isoform promotes cell proliferation of human neural stem cells in a concentration-dependent manner [39]. Dexmedetomidine (DEX) protected PC12 cells from ROS-induced cytotoxicity via inhibition of collagen alpha-1(III) chain expression and mitogen-activated protein kinase (MAPK) pathway activation [40,41]. DEX-mediated neuroprotection is associated with a dose-dependent restored impaired proliferation of PC12 cells in a stress condition, as reflected by the increased cell viability, which were consistent with the decreased expression of tumor suppressor protein p21 and increased expression of cell cycle-related cyclin D1 [42]. Interestingly, these data indicate that neuroprotective effects of β-scoliidine against H_2_O_2_-induced oxidative stress could be explained by the increased cell proliferation in PC12 cells. Despite that, more studies are needed to better clarify the mechanism underlying how β-scoliidine induces cell proliferation, and its relation to the neuroprotective effect.

## Conclusions

Comprehensive LC-MS and MS/MS analyses of the crude venom extract from the solitary scoliid wasp *Scolia decorata ventralis* revealed the component profile of this venom. The two major peptide components, α-scoliidine and β-scoliidine, are bradykinin-related peptides. β-scoliidine showed an effective cytoprotective effect, probably due to the observed increase in the cell number. This is the first case for solitary wasp venom peptides to show neuroprotective activity.
